# Magnetic field and nuclear spin influence on the DNA synthesis rate

**DOI:** 10.1038/s41598-022-26744-4

**Published:** 2023-01-10

**Authors:** Sergey V. Stovbun, Dmitry V. Zlenko, Alexander A. Bukhvostov, Alexander S. Vedenkin, Alexey A. Skoblin, Dmitry A. Kuznetsov, Anatoly L. Buchachenko

**Affiliations:** 1 N.N. Semenov Federal Research Center for Chemical Physics, RAS, Kosygina 4, Moscow, Russia 119991; 2 A.N. Severtsov Institute of Ecology and Evolution, Lenin avenue 33, Moscow, Russia 119192; 3 N.I. Pirogov Russian National Research Medical University, Ostrovityanova 1, Moscow, Russia 117997; 4 Institute of Problems of Chemical Physics RAS, Akademika Semenova avenue 1, Chernogolovka, Moscow Region Russia 142432; 5 Scientific Center of the Russian Academy of Sciences, Lesnaya 9, Chernogolovka, Moscow Region Russia 142432; 6 M.V. Lomonosov Moscow State University, faculty of Physics, Lenin Hills 1/2, Moscow, Russia 119192

**Keywords:** DNA, Enzyme mechanisms, Physics

## Abstract

The rate of a chemical reaction can be sensitive to the isotope composition of the reactants, which provides also for the sensitivity of such “spin-sensitive” reactions to the external magnetic field. Here we demonstrate the effect of the external magnetic field on the enzymatic DNA synthesis together with the effect of the spin-bearing magnesium ions ($^{25}$Mg). The rate of DNA synthesis monotonously decreased with the external magnetic field induction increasing in presence of zero-spin magnesium ions ($^{24}$Mg). On the contrary, in the presence of the spin-bearing magnesium ions, the dependence of the reaction rate on the magnetic field induction was non-monotonous and possess a distinct minimum at 80–100 mT. To describe the observed effect, we suggested a chemical scheme and biophysical mechanism considering a competition between Zeeman and Fermi interactions in the external magnetic field.

## Introduction

The magnetic field can affect the biological processes in various aspects, that was summarized in the very recent review by Zadeh-Haghighi and Simon^1^. In particular, the static magnetic field of significant induction ($9.4\,T$) inhibits the DNA synthesis in mice, therefore, suppressing the tumor growth and even affecting the normal tissues, for example, decreasing the amount of blood cells^2^. On the other hand, the inhibition of apoptosis by the magnetic field can cause an increase in the risk of cancer development^3, 4^. However, the latter effect is often considered to be related to the reactive oxygen species production rather than DNA synthesis^4^. Besides that, DNA fragmentation was registered under extremely low-frequency electromagnetic field action in human cells^5^. The latter results provide the pharmacological basis of magnetic field usage in anti-tumor therapy. Meanwhile, many works report no effect of static magnetic field on the DNA and RNA synthesis rates^6^. Such contradictory results are quintessential for complex living systems.

The rates and pathways of chemical reactions can depend on the properties of nuclei, both their mass and their spin. Nuclear mass selectivity of chemical reactions is well known and results in the Classical Isotope Effect^7^. The Magnetic Isotope Effect (MIE) phenomenon is the dependence of chemical reaction rate on the nuclear spin and nuclear magnetic moment of the reactants^8^. Since the discovery of $^{13}$C MIE in the reaction of dibenzyl ketone photolysis^9^, it was described for several other elements, such as, for example, sulfur^10^ and germanium^11^. The first example of the MIE manifestation in biochemistry was found in creatine kinase inhibition reaction by methyl-mercury chloride^12^. The interaction of methylmercury chloride with creatine kinase appeared to be an ion-radical spin-selective process sensitive to the electron spin, which inevitably led to the nuclear spin selectivity.

Later MIE was demonstrated in mitochondria for enzymatic phosphorylation in the presence of different magnesium ions^13^. In the presence of the magnetic (bearing non-zero spin) ions ($^{25}\hbox {Mg}^{2+}$, $^{67}\hbox {Zn}^{2+}$, or $^{43}\hbox {Ca}^{2+}$), the rate of enzymatic phosphorylation was two to four times greater than in the case of their non-magnetic counterparts^8^. Moreover, the rates of the reaction were equal in presence of ions having different masses and equal spin, which excluded the classical isotope effect manifestation. The magnetic isotope effect was also revealed in the reaction of DNA synthesis^14^. Complete substitution of the natural magnesium ions pool ($^{24}$Mg:$^{25}$Mg:$^{26}$Mg $\sim$ 8:1:1) with the paramagnetic ones decreased the DNAPol$\beta$ catalytic activity^14^. The effect was strong enough to provide for a pronounced anti-proliferation effect on HL-60 human acute myeloid leukemia cells^15, 16^.

Alongside the classical MIE, the MIE-demonstrating reactions were found to be sensitive to the external magnetic field^13^. In particular, the external magnetic field (of 50–90 mT) significantly increased the amplitude of classical MIE. However, the latter results were not confirmed due to the contamination of the specimens with spin-bearing iron ions^17^. The influence of the external magnetic field differed for the creatine-kinase specimens loaded with pure $^{25}\hbox {Mg}^{2+}$ and $^{24/26}\hbox {Mg}^{2+}$^13^. In the former specimens, the rate of ATP synthesis grew with the external magnetic field induction, while in the latter, it decreased, but the amplitude of the effect was significantly lower. Such a bidirectionality of the external magnetic field influence was not explained. It is not clear if the supposed scheme, comprising only a single-electron transfer from phosphate oxygen to the magnesium ion^13^ and explaining the mechanism of the MIE origin, can also explain the influence of the external magnetic field. Indeed, the bidirectionality of the external magnetic field influence may require some complications of the scheme, for example, an additional ion-radical pair participating in the reaction.

In this work we analyze the biophysical aspect of the interaction of an unpaired electron spins with a weak (less than 100 mT) external magnetic field trying to verify if the supposed earlier scheme is sufficient for the description of obtained experimental results. One of the sufficient peculiarities of the effects under discussion is a elative weakness of the impact of the magnetic field, as the energies of interaction of such a weak fields with the electron ($\mu H$) are hundreds of thousands of times smaller than thermal energies ($k_BT$), which rises a problem of the origin of the effect^1^. Available at the moment, empirical data was rather poor, so we have conducted several experiments with DNA polymerase $\beta$^18^ specimens placed in the permanent magnetic field of precisely measured induction. The external magnetic field affected the rate of DNApol$\beta$ but decreased its activity rather than increased it.

## Results and discussion

The dependencies of the DNApol$\beta$ activity on the permanent magnetic field induction drastically differ according to the magnetic properties of the magnesium ions. In $^{24}\hbox {Mg}^{2+}$ loaded samples, the catalytic activity monotonously decreased with the magnetic field induction, reaching a level of about 30 % of the initial one at 150-200 mT. On the contrary, in the $^{25}\hbox {Mg}^{2+}$ loaded samples, the magnetic field dependence was non-monotonous and possessed a minimum. The catalytic activity of DNApol$\beta$ in $^{25}\hbox {Mg}^{2+}$ loaded samples monotonously decreased to the level of about 20 % of the initial one at approximately 100 mT, and then grew up and restored to the level of about 85 % above 200 mT.

**Figure 1:**
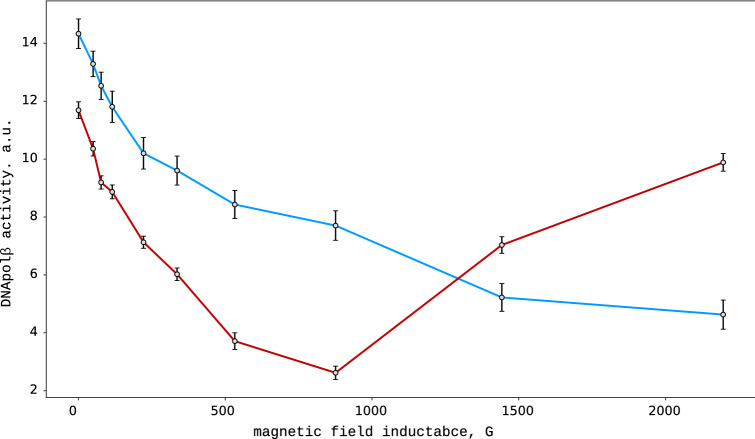
Catalytic activity of DNApol$\beta$ as a function of the external magnetic field induction. The blue curve corresponds to the samples loaded with the non-magnetic $^{24}\hbox {Mg}^{2+}$, while the red one – to the samples containing only magnetic magnesium isotope ($^{25}\hbox {Mg}^{2+}$). The error bars reflect the standard deviation calculated over three independent repeats.

**Figure 2:**
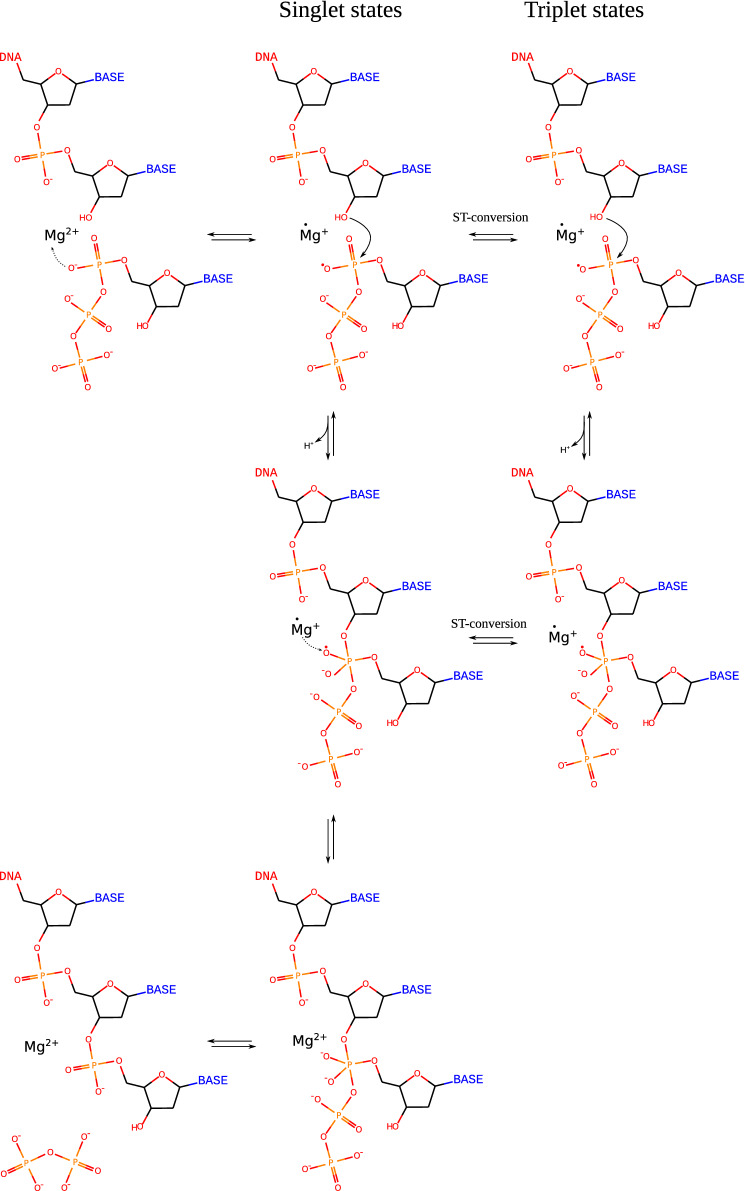
Principal scheme of the ion-radical mechanism supposed to describe the origin of the magnetic isotope effect in the reaction of the DNA synthesis by DNApol$\beta$ enzyme. The electron transfer is marked by dotted arrows, while the nucleophilic attack – by the plain ones. The scheme was reproduced from Buchachenko et al., 2012^8^ with modifications.

To explain the magnetosensitivity of the DNA synthesis rate, some ion-radical pair should be supposed^19^, however, the problem of such a pair determination looks quite challenging and rises a lot of questions^1^. Nevertheless, the ion-radical pair providing for the magnetosensitivity of the enzymatic ATP synthesis was selected^8^. A key step was proposed to be an electron transfer from the oxygen of phosphate to the magnesium ion (Fig. 2). Such a reaction does not occur in solution, but in the protein-DNA surrounding, water molecules are squeezed out from the hydration shell of the $\hbox {Mg}^{2+}$^20^. Quantum chemistry calculations demonstrate a significant growth in the electron affinity of $\hbox {Mg}^{2+}$ ion with a decrease in number of water molecules in hydration shell^21^. Therefore, the electron transfer from phosphate oxygen to magnesium ion became theoretically non-forbidden. The generated ion-radical pair occupies a singlet state and can easily recombine through the back electron transfer, which decreases the rate of the reaction. However, the ion-radical pair can undergo a singlet-triplet (ST) conversion, significantly simplified by a hyperfine interaction between the magnetic moment of the unpaired electron and nuclear spin of $^{25}\hbox {Mg}^{2+}$ or external magnetic field (the right branch of the scheme). In the triplet (T) state, the back electron transfer and recombination of the ion-radical pair become spin-forbidden, while other reactions can proceed precisely in the same way as in a singlet (S) state (left branch of the scheme).

Providing for the ST-conversion, the magnetic field, in fact, provides for an increase in T-states’ concentration, as they were absent at the ion-radical pairs’ generation. As far as backward recombination is forbidden in T-state, ST-conversion should increase the rate of the reaction. On the other hand, the back electron transfer and recovery of the non-radical state of the enzyme is also forbidden in T-state. Therefore, if the enzyme recovery in the last stage (Fig. 2) became hindered, the reaction rate can decrease in a magnetic field or at elevated spin-bearing ions concentrations.

The main point that should be discussed is the suitability of the scheme (Fig. 2) to the results obtained in experiments with DNApol$\beta$ (Fig. 1). We have to disclaim that the exact nature of the ion-radical pair is a highly disputable problem^1^. However, our experiments revealed the sensitivity of the reaction to the magnesium isotope composition. Therefore, one has to conclude magnesium ion should participate in that ion-radical pair formation. Moreover, a relative weakness of the nuclear magnetic moments implies the magnesium ion should be one of the atoms bearing unpaired electron. Otherwise, the sensitivity to isotope composition and external magnetic field would not be so prominent. Magnesium ion cannot be the electron donor, so we have to suppose it is an acceptor. The most probable candidate for the electron donor is the oxygen atom of phosphate group. Here are two reasons: 1. The $\pi$-electrons are highly delocalized inside the phosphate anion, decreasing the free energy of the oxidized radical state; 2. Such an electron transfer increases a partial positive charge on the phosphorous atom, which simplifies the nucleophilic attack of 3’-oxygen on it, which results in DNA chain synthesis. Nevertheless, regardless of the chemical nature of the ion-radical pair, our reasoning does not fade in case the real mechanism differs from the one described in Fig. 2. Moreover, both increase in $^{25}$Mg concentration and application of the external magnetic field promote the T-states’ generation. Therefore, regardless of the details of the mechanism, one has to conclude the T-states’ generation decreases the rate of the DNA synthesis at least by DNApol$\beta$.

First of all, we have to make a strong supposition. In general, the spin subsystem is weakly coupled with other parts of the system, especially in the case of the soft matters as proteins are. Therefore, the coherence lifetime of the spin states ($\tau _s$) is quite long and comprises about $10^{-6}$–$10^{-7}$ sec^9^. On the other hand, the electron transfer can occur only while the phosphate group and magnesium ion are close enough to each other and such a state exists for a long enough time. Using a quasiclassical approach^22^, the characteristic time of electron transfer ($\tau _t$) over the distance *R* can be written as:

$$\begin{aligned} \tau _t = \frac{1}{\nu } e ^ {R/a} \end{aligned}$$

where $\nu$ is a frequency of electron oscillations ($\sim 10^{16}$ Hz according to the Bohr atom model), and *a* is an electron localization radius in phosphate anion equaling approximately to the radius of the oxygen atom ($\sim 0.15$ nm). Despite the electron delocalization effect, the radius of electron localization rarely reaches the values of 0.2 nm or greater^22, 23^. Given that the reagents are squeezed in the cavity of the reaction center inside the polymerase globule, the distance *R* cannot exceed several nanometers. Therefore, for the distance of 2–3 nm, the characteristic time of electron transfer $\tau _t$ would be equal to $10^{-10}$–$10^{-8}$ sec, which is much smaller than the coherence lifetime of the spin states ($\tau _s \sim 10^{-6}$–$10^{-7}$ sec^9^). Therefore, the electron transfer can really occur within the specified spatial and temporal frames. Thus, the dissipation can be neglected for the spin subsystem if the processes under consideration are faster. All of the reasonings below were made following this supposition.

In the scheme (Fig. 2), the critical point is an electron transfer from the oxygen of the phosphate ion to the magnesium ion. As far as the initial state was a singlet, the resulting ion-radical pair will also be a singlet due to the angular momentum conservation law. In the case of zero-spin ions ($^{24}\hbox {Mg}^{2+}$), a conversion between S and $\hbox {T}_0$ states became possible under the external magnetic field action due to the difference in g-factors of the radicals. Moreover, the rate of this process increases with the megnetic field inductance, most probably, providing for the decrease in the DNA synthesis rate.

Substitution of zero-spin $^{24}$Mg with spin-bearing $^{25}$Mg (having a magnetic moment $\mu ^* = -0.855\,\mu _n$, where $\mu _n$ is a Bohr magneton) qualitatively changes the DNApol$\beta$ behavior in a magnetic field (Fig. 1). Instead of the monotonous decrease of the reaction rate with the increase of the field induction, the enzyme activity decreases and reaches the minimum at $H_{min} = 80$–100 mT. The observed non-monotonous dependence denotes some reorganization in the spin-system occurring at 80–100 mT. Note that the spin-switching processes can take place in DNA, which can even be used for logical elements’ construction^19^. The decrease in the enzyme activity, in this case, was more prominent as compared to the $^{24}$Mg loaded samples (Fig. 1). This effect can be related to the synergy of S$\rightarrow \hbox {T}_0$ and S$\rightarrow \hbox {T}_+/\hbox {T}_-$ conversions due to the interaction with the external magnetic field and the spin of $^{25}$Mg nuclei, correspondingly. A further increase in the magnetic field induction results in the growth of β the enzyme activity. The threshold magnetic field intensity of 80–100 mT corresponds to the energy of Zeeman interaction $\varepsilon = \mu ^* H_{min} = 0.46$–0.56 J/mole. The latter is close to the energy of the interaction of electrons and nuclear spins, providing for Fermi hyperfine splitting^24^. Therefore, it is reasonable to propose break-up in the dependence of the reaction rate on the inductance is related to the Fermi splitting, as the latter can effectively suppress S$\rightarrow \hbox {T}_+/\hbox {T}_-$ conversion.

Resuming the reasonings given above, the magnetosensitivity of both $^{24}$Mg and $^{25}$Mg loaded samples to the external magnetic field is provided by T-states production induced by either Zeeman, or Fermi interactions. In the samples loaded with $^{24}$Mg, the S$\rightarrow \hbox {T}_0$ conversion is provided by Zeeman interaction. On the contrary, in the $^{25}$Mg loaded samples, the S$\rightarrow \hbox {T}_+/\hbox {T}_-$ conversion is induced by Fermi hyperfine electron-nuclear interaction, which effectively functions in zero magnetic field. However, magnetic field of inductance $H_{min}$ compensates the Fermi interaction and hindering ST-conversion. Such a competition between Zeeman and Fermi interactions was quantitatively described in the frame of irrefutable theory by McLauchlan and Steiner^25^.

Summarizing the work, we should repeat that the supposed ion-radical intermediate can easily recombine recovering the initial compounds. The probability of this reaction seems to be relatively high and it seems to be crucial for the overall reaction rate. In case for some reason the recombination rate decreases, the rate of DNA synthesis should increase and vice versa. As far as the backward recombination is spin-prohibited in the T-states of the ion-radical intermediate, the overall reaction rate should increase with the intensification of ST-conversion. On the other hand, the regeneration of the enzyme by the recombination of the radicals is also spin prohibited in T-states (Fig. 2). This implies an opposite effect of decrease in the reaction rate with ST-conversion intensification. Therefore, the supposed scheme allows the effect to have different signs, depending on the peculiarities of protein spatial structure. According to the scheme, we can suppose the sign of the effect would be determined by the relation between the probabilities of the ST-conversion at the first and the last steps. Such an ambivalence allows description of the decrease in the DNA synthesis rate and increase in the ATP synthesis rate under weak magnetic field in the frames of a single hypothesis.

## Methods

### Chemicals

$^{25}\hbox {MgCl}_2$ (88.6 % $^{25}$Mg isotopic enrichment, A grade) and the $^{24}\hbox {MgCl}_2$ (92.4 % $^{24}$Mg isotopic enrichment, A grade) were purchased from the Obninsk Institute of Medical Radiology RAS (Obninsk, Russia). The tritium-labeled nucleotide was purchased from New England Nuclear, USA.

### Cell culture

The HL-60 human myeloid leukemia cell line has been purchased from the Hungarian Cell Bank, Paseur Institute of Hungary, Szeged, NCBI Code C42. Cells were maintained in suspension culture at $+37\,^\circ$C under 5 % $\hbox {CO}_2$/air in RPMI 1640 (Gibco, UK) supplemented with 10 % FCS and antibiotics: 100 U/ml of Penicillin and $100\,\mu$g/ml of Streptomycin. The cells were subcultured three times weekly, ATRA (Sigma, USA). This procedure has been originally adopted by Olins et al.^26^ and then modified by Roy et al.^27^.

### Enzyme isolation

$\beta$-like DNA polymerases (DNApol$\beta$) were isolated from HL-60 cells. An original method included treatment with phenol-chloroform, precipitation with ammonium sulfate, and subsequent gel filtration^18^. The specimens were electrophoretically homogenous. The catalytic activity of DNApol$\beta$ was evaluated by the intensity of tritium label ([$^3$H]dTTP) incorporation into newly synthesized DNA strands: counts per minute of incubation under optimal conditions, per 1 mg of the pure enzyme^18^. Protein and DNA quantitative microcolorimetric measurements as well as enzyme SDS-PAGE (0.5/10 %) electroforetic homogeneity/purity patterns were carried out and monitored as was described earlier^28^.

DNA-Pol$\beta$ catalytic activity values were expressed in amounts of [$^3$H]dTTP incorporated into nascent DNA chains in 1 min of incubation at optimal conditions corrected per 1.0 mg of pure enzyme ([$^3$H]DNA cpm/mg protein) as was described earlier^16, 18^.

### Experimental protocol

The samples of DNApol$\beta$ solutions ($30\,\mu$g/ml) in plastic Petri dishes (30 mm in diameter) were arranged in a stack and placed on the face of the cylindrical Nd magnet (N38, $60 \times 30$ mm). The magnetic field induction was not uniform and decreased with the distance to the magnet’s central axis. According to the direct measurement, the difference in magnetic field inductance between the center and edge of the Petri dish never exceeded 10 %. This allowed us to dismiss the non-uniformity and plot the dependencies against the inductance found on the central axis of the magnet. The samples were incubated for 60 min at $37\,^\circ$C. In such a way, the distance between the dishes (10 mm) provided for th gradual decrease of the magnetic field induction, that was directly measured (the values obtained can be found in Fig. 1). The magnetic field induction was measured using an in-house magnetometer based on a linear-output Hall sensor (AD22151, Analog Devices, USA)^24^, providing for the accuracy of about 0.35 mT. The obtained values were in a good agreement with theoretical predictions. Therefore the incubation at different magnetic field inductions was performed simultaneously, ensuring the uniformity of the conditions and comparability of the obtained results. Three independent repeats were made for whole set of the different magnetic field inductions. Given that each experiment included all the range of the magnetic field inductions, three independent curves were obtained, rather than individual measurements at each magnetic field induction.

## Data Availability

All data generated or analyzed during this study are included in this published article.
