# Author Correction: Magnetic field and nuclear spin influence on the DNA synthesis rate

**DOI:** 10.1038/s41598-023-31317-0

**Published:** 2023-03-14

**Authors:** Sergey V. Stovbun, Dmitry V. Zlenko, Alexander A. Bukhvostov, Alexander S. Vedenkin, Alexey A. Skoblin, Dmitry A. Kuznetsov, Anatoly L. Buchachenko

**Affiliations:** 1 N.N. Semenov Federal Research Center for Chemical Physics, RAS, Kosygina 4, Moscow, Russia 119991; 2 A.N. Severtsov Institute of Ecology and Evolution, Lenin Avenue 33, Moscow, Russia 119192; 3 N.I. Pirogov Russian National Research Medical University, Ostrovityanova 1, Moscow, Russia 117997; 4 Institute of Problems of Chemical Physics RAS, Akademika Semenova Avenue 1, Chernogolovka, Moscow Region Russia 142432; 5 Scientific Center of the Russian Academy of Sciences, Lesnaya 9, Chernogolovka, Moscow Region Russia 142432; 6 M.V. Lomonosov Moscow State University, Faculty of Physics, Lenin Hills 1/2, Moscow, Russia 119192

Correction to: *Scientific Reports* https://doi.org/10.1038/s41598-022-26744-4, published online 10 January 2023

The original version of this Article contained an error in the spelling of the author Alexander S. Vedenkin which was incorrectly given as Alexander A. Vedenkin.

The original Article has been corrected.

